# NMR-Fragment Based Virtual Screening: A Brief Overview

**DOI:** 10.3390/molecules23020233

**Published:** 2018-01-25

**Authors:** Meenakshi Singh, Benjamin Tam, Barak Akabayov

**Affiliations:** Department of Chemistry, Ben-Gurion University of the Negev, Beer-Sheva 8410501, Israel; meenaksh@post.bgu.ac.il (M.S.); tamb@post.bgu.ac.il (B.T.)

**Keywords:** fragment-based drug discovery, nuclear magnetic resonance, fragment based virtual screening

## Abstract

Fragment-based drug discovery (FBDD) using NMR has become a central approach over the last twenty years for development of small molecule inhibitors against biological macromolecules, to control a variety of cellular processes. Yet, several considerations should be taken into account for obtaining a therapeutically relevant agent. In this review, we aim to list the considerations that make NMR fragment screening a successful process for yielding potent inhibitors. Factors that may govern the competence of NMR in fragment based drug discovery are discussed, as well as later steps that involve optimization of hits obtained by NMR-FBDD.

## 1. Introduction: Fragment Screening as a Central Approach in Lead Molecule

A lead molecule is usually defined as a small molecule with a molecular weight (MW) of approximately 500 Da, which can bind its target through H-bonds with no more than five hydrogen bond donors and no more than 10 hydrogen bond acceptors, is flexible enough with rotatable bonds to allow functional binding to the target, and favorably lipophilic with partition coefficient (cLogP, a measure of hydrophobicity) less than 5. All these properties form the basis to develop a lead molecule into a promising drug candidate [1,2], an early step in the process of translating small molecules into medicines. Subsequent steps in a lead-to-drug process usually involve optimization cycles by synthesis of structurally related analogs and activity related measurements. 

**Figure 1:**
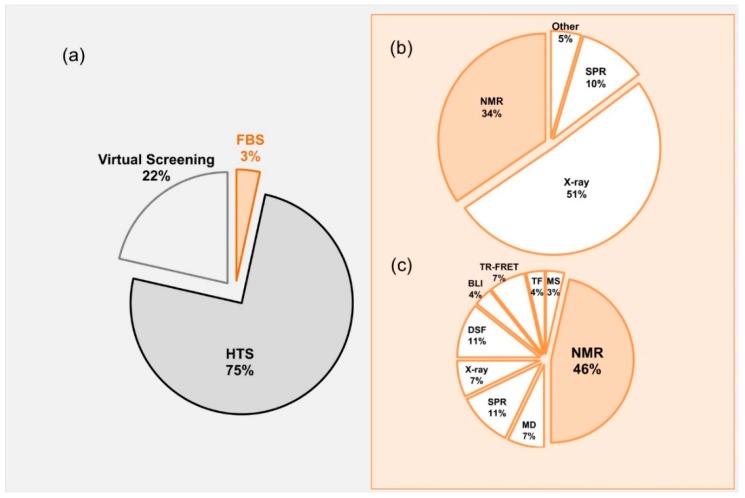
(**a**) Pie-chart representing contributions of different techniques in Drug Discovery. The numbers obtained by a PubMed search of keywords containing terms referring to high-throughput screening, fragment-screening, and virtual screening focusing only in research articles; (**b**) Approved FDA drugs from 2017 and development methods. The information was collected from KEGG-DRUG website (http://www.genome.jp/kegg/drug/br08319.html?id=D01441); (**c**) Values representing pubmed entries and published for 2017. NMR, Nuclear magnetic resonance; MD, Molecular dynamics; MS, Mass Spectrometry; SPR, surface plasmon resonance; DSF, differential scanning fluorimetry; BLI, biolayer interferometry; X-ray, Crystallography.

Lead molecules have been discovered mainly through identification of active compounds by screening of large chemical databases. Along with high throughput screening (HTS) and virtual screening, fragment based screening (FBS) has been established as a central approach in finding the initial “Hits” that can readily be developed into “Leads” [3,4,5,6,7,8] (Figure 1). Most of the published data related to drug discovery is originated from HTS, whereas FBS contributes only minor portion (3%) of the published data (Figure 1a). Approved FDA drugs that were originated from FBDD where mainly developed using crystallography or NMR (Figure 1b), however, in 2017 most of the development was using NMR.

The idea behind FBS is to test fragment molecules that are small enough, thus covering a larger chemical space [9,10]. FBS generally offers higher hit rates and binding efficiencies compared with HTS [11,12]. Nevertheless, due to their small size, fragment hits are usually weak binders and must be developed into higher affinity larger molecules in order to be ultimately developed into a lead molecule. HTS and FBS have been considered as complementary approaches in drug discovery. Some of the pharmaceutical and biotech industries used FBDD in conjunction with HTS that shorten the early phase of the drug discovery process providing robust lead compound series [13]. Wu et al. described the advantages of FBDD and HTS approaches in a screening strategy designated as HTS by NMR, presenting ligand discovery by fragment-based approach. The approach combines basic combinatorial chemistry principles with NMR spectroscopy to screen larger libraries of compound fragments [14].

Targets can be screened by HTS only if the inhibition they induce can produce an obvious readout. Thus, biochemical and cell based assays used in HTS screening are usually not suitable to detect the weak binding interactions of fragment molecules to their macromolecule target. Variety of biophysical binding techniques were adapted to detect weak interactions between fragment molecules and their targets [15]. Since the pioneering study published in 1996 by Shuker et al. [8], NMR has become the most popular technique for application in FBS, as it can detect weak binding between the fragment and target macromolecule, with a KD in the low mM-range [16].

Since the early 1990s, advances in data acquisition techniques, combinatorial chemistry, high-throughput screening approaches, genome sequencing, short-interfering RNA (siRNA) tools and gene expression profiling [17] have helped to design and optimize drugs for the pharmaceutical industry [18]. In particular, high-throughput screening (HTS) became a dominant approach for the discovery of hit molecules [19]. By the early 2000s, companies were building multimillion compound libraries, which were the source for many current clinical candidates [20]. However, when screened against novel or more difficult targets the vast majority compound libraries sometimes yielded few hits (<1%) or, in more problematic cases, yielded hits that were false positives [21,22]. Examples for difficult or novel targets can be protein-protein interactions [23] and range of targets outside the ribosome, the cell wall synthesis and DNA gyrase that comprise clinical targets for the most successful antibiotics [24]. In fact, an estimated number of combinations of the spatial arrangement of atoms in a drug-like molecule (MW of 500 Da) in a standard HTS library is 10^60^, and the chemical space each molecule covers is therefore very limited [25,26] and thus results in a low hit rate for HTS. Compared with small fragment molecules, drug-like molecules possess functional groups that may pose more steric hindrance or electrostatic repulsion in a binding site [27,28].

A variety of mechanism-based assays for HTS that are mechanism-based were described in the literature. One such case for a broad mechanism to identify small molecules for cardiovascular disease was described that can provide high content phenotypic screening [29]. Phenotypic high-throughput drug screens, also called chemical genetic or in vivo screens, investigate the ability of individual compounds from a small molecule collection to inhibit a biological process or disease model in living cells or intact organisms. A protease cleavage assay would be another specific example for mechanism based HTS assays [30]. This assay used cellular FRET based methods, developed using fluorescent proteins or dyes linked by a protease. Cleavage of the probe is measured by a change in fluorescence upon activation of proteases such as caspase-3 [31] and hepatitis virus (HCV) NS2/3 [32]. In addition, HTS largely depends on the development of a good, usually one-step primary assay that yields readout of a biochemical/cellular reaction. As a consequence, targets that don’t form a readout may be considered mistakenly as non-druggable or may not gain proper recognition as a “bona fide” drug target. Therefore, further improvements in drug discovery have directed researchers’ attention toward defining druggable targets and developing a more rational and focused approaches, concentrating on the quality, rather than the quantity of hits and leads.

Although combinatorial chemistry had struggles in its earlier days, there are few lead molecules that came from the HTS compared to other approaches. However, a significant number of drugs in clinical trials originated from HTS campaigns, validating HTS as a bona fide mechanism for hit finding [33]. In the recent years FBDD has emerged as a major approach of hit to lead discovery in research of human diseases, where conventional approaches in drug discovery have failed [34].

**Figure 2:**
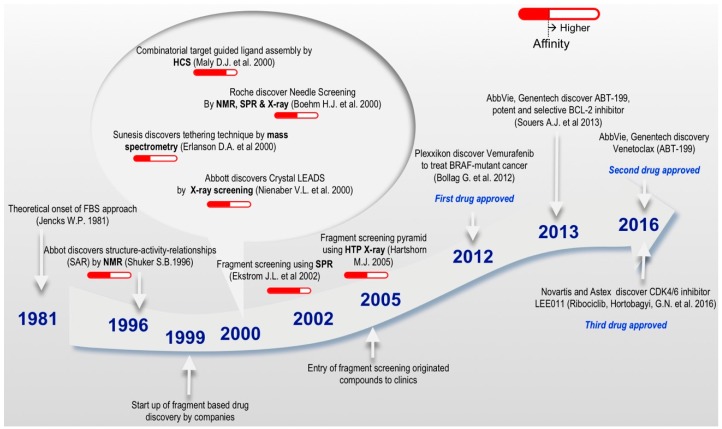
Timeline—selected landmarks which significantly influenced the development of FBDD. The range of affinity describes detection of compound binding to macromolecular target. Solid Red colored bars represent weak affinity in the range of 100 µM–10 mM and hollow red colored bars represent strong affinity in the range of 1 µM–100 µM. The development of FBDD was started in 1981 by Jencks W.P. [35] and then in 1996 ‘SAR by NMR’ by Shuker S.B. et al. [8]. Most of the discoveries occurred in 2000 among which Erlanson D.A. et al. discovered Tethering techniques [43], Maly D.J. et al. used combinatorial target guided ligand assembly [44], Boehm H.J. et al. discovered needle screening [45] and Nienaber V.L. et al. discovered crystal leads [46]. In 2002 Ekstrom J.L. et al. used fragment screening by SPR [47] and in 2005 Hartshorn M.J. et al. used fragment screening by X-ray crystalloraphy [48]. Bollag G. et al. discovered the first approved drug Vemurafenib in 2012 [49], Souers A.J. et al. discovered ABT-199, a potent selective BCL-2 inhibitor in 2013 [50], and Hortobagyi G.N. et al. discovered recently the third approved drug LEE011 in 2016 [51].

The concept of drug design has gained much attention, especially with the progression of the fragment-based philosophy (Figure 2) over the past 20 years. Fragment linking is one of the powerful ways to develop a fragment hit into a lead compound. The concept was first introduced by Jencks in 1981, based on the theory of additivity of binding free energies with the idea that large molecules can be considered as the combination of two or more fragments that contain all the features necessary for binding to the target protein. Hence, linked molecules with micromolar affinities can be obtained from fragments that bind in the millimolar range, a central principle presented in the onset of FBS approach [35]. FBS is a rapid and economic alternative to HTS [36] and has been established as a mainstream strategy to discover novel high-quality drug-like molecules in both industry and academia [34,37,38,39,40]. FBS adds novelty and flexibility to lead molecule generation proficiencies and increases the probability of success in lead molecule development [41]. FBS takes a different approach to that of HTS and virtual screening; instead of screening libraries of million compounds to find drug-sized hit molecules, FBS begins with limited collections of low MW compounds (150–300 Da). Unlike HTS where the readout is usually based on a biochemical process of the target macromolecule using a functional assay, FBS monitors the binding of small molecules with high binding energy per atom to the its binding partner (ligand efficiency) [27]. Thus, development of a biochemical or cell-based assay is not required in FBS; instead the direct binding of small molecules to the target macromolecule is monitored.

Fragment molecules are usually defined as functional chemical groups with less than 20 non-hydrogen (or ‘heavy’) atoms. Such small molecules present low complexity, owing to their low MW, which allows an efficient exploration of diversified chemical space [42]. The small molecule size brings binding flexibility, as the fragment molecule can bind to various loci of a target in several ways. Although the binding of additional fragment to the same site brings additional intrinsic binding energy to the target [35], the transition from fragments into lead molecules constitutes the bottleneck in FBS. Nevertheless, more and more options to overcome these time and resource consuming problems are becoming available, and several molecules developed using this method have been approved by the FDA (Figure 2) or are being studied in clinical trials [34].

This review describes the identification of fragment molecules and their optimization steps into lead-molecules, specifically focusing on NMR as a specialized tool adapted for FBS and optimization. The principles behind the strategic approach of FBS are also discussed in comparison with other available technologies for screening. A survey on the current design of small molecule libraries adapted for NMR screening is presented. Finally, the impact of FBS on the development of candidate molecules in the current drug development pipeline and future directions of FBS are discussed.

## 2. Detection Methods Used for FBS

The binding of fragments is often very weak, and therefore biophysical techniques with high detection sensitivity such as NMR [52,53,54], SPR [55], microscale thermophoresis (MST) [56], capillary electrophoresis [57], weak affinity chromatography [58], biolayer interferometry/ultra-filtration [59], native mass spectrometry [60], isothermal titration calorimetry [61], and X-ray crystallography [62] are used to monitor the binding interactions. Although X-ray crystallography offers the most detailed delineation of protein–ligand binding modes, its application in primary FBS has been limited thus far. Since high concentrations of fragments are necessary to compensate weak binding (high K_D_) in protein pockets, the concentration of the fragment molecules is limited by the aqueous solubility [63]. In addition, in X-ray crystallography the target macromolecule needs to be crystalizable alone and with the fragment molecule. Although many new technologies have been developed over the past decade, NMR based screening of small molecules is advantageous, as it presents high sensitivity for weakly-bound target-ligand complexes [64,65,66]. NMR measures in solution, does not require any modification of the molecule and therefore is best suited for FBS [64,65,66].

Computer-aided techniques are also used for fragment screening, for example molecular docking. Molecular docking is the generation of hypothetical computer-guided protein–ligand complexes as a means for understanding the mechanism of action or as a starting point for structure-based ligand optimization. The application of docking to predict binding of small molecules remains a challenge due to the following reasons: (1) fragment molecules are small in size and have low MW, as a result, a number of interaction sites on protein surfaces (closely related energy minima) might be found to theoretically accommodate the fragment binding, which would lead to false docking positions. Even if fragments are placed into the correct pocket, if the binding pocket is large, it still might result in incorrect binding modes [67]; (2) fragments usually have weak target affinities than drug-like molecules with higher K_D_ values of over than 3 orders of magnitude i.e., in a range of (μM–mM) [68], therefore scoring functions are not always accurate enough to predict the binding modes of fragments [69].

However, instead of empirical scoring functions, Shoichet and co-workers used physics-based scoring function to prioritize active fragments [70]. They employed in silico fragment screening to find AmpC β-lactamase inhibitors [70] and used a total of 137,639 fragment molecules from the ZINC database, docked into an apo AmpC crystal structure (PDB ID code 1KE4) using the docking software DOCK3.5.54. Among the hits obtained, forty-eight top ranked fragments were subjected to an in vitro enzyme inhibition assay and 23 molecules with K_i_ values in the range of 0.7–9.2 mM were identified. Although the K_i_ values present low potency of inhibition, the inhibitors are presumed to be specific. Moreover, this in silico FBS study yielded a higher hit rate (48%) than both virtual screening and HTS of drug-sized molecules due to better coverage of chemotypes at the fragment level.

In another example, Caflisch and colleagues developed a fragment-based procedure, called anchor-based library tailoring (ALTA) used for docking of large libraries of compounds to find inhibitors for EphB4 tyrosine kinase [71]. ALTA starts by decomposition of the compound library into rigid fragments followed by docking and ranking of the fragments. In addition, they have used pharmacophore constraints to preselect compounds for docking which adeptly reduced the library size. Optimization as a follow up step after FBS is therefore beneficial for bringing fragment molecules into the desired size and efficacy.

Computational methods have been developed to identify and characterize hot spots for fragment binding [72]. However, binding modes and computation of free energies by docking experiments are not yet sufficiently accurate to correctly predict early structure-activity relationships (SAR) around weak-affinity fragments [73]. Despite the success of many examples of docking in FBDD, applying docking in FBDD remained challenging because of possible promiscuous binding modes, the lack of handles to fit fragments into the pocket, and biases in docking scoring functions [74,75,76,77].

## 3. Design of Fragment Libraries

One important consideration for the screening setup is the choice of fragment libraries designed for FBDD [78]. The most common fragment libraries, designed for screening against an extensive variety of targets, are diverse sets of compounds with high pharmacophore diversity or physicochemical properties such as molecular mass, lipophilicity etc. [79]. The molecules are filtered to remove functional groups that may contribute to additional chemical reactivity, toxicity, and false positives [22,80,81,82,83].

Molecules in HTS libraries fulfill Lipinski’s “rule of five” [84], which occasionally enforces researchers to compromise on the disposition properties (absorption, distribution, metabolism, and excretion, ADME) to obtain potent inhibitors. By analogy to the Lipinski’s “rule of five”, molecules in libraries adapted for FBS obey the “rule of three” (Ro3) [85], in which a molecule has: (1) a molecular weight ≤300 Da; (2) a hydrophilicity value, clogP ≤ 3; (3) number of hydrogen bond donors and acceptors ≤3; (4) number of rotatable bonds ≤3; and also, (5) to a lesser extent, a molecular polar surface area ≤60 Å^2^. The Ro3 proposed by Astex [85] has been widely applied to design fragment screening pools. Fragments that are screened in combination with X-ray crystallography or NMR are usually well soluble (e.g., can reach to 25–200 mM) in aqueous buffer and structural information is available for further optimization of the hits [48,86].

However, the simplicity of fragments of Ro3-compliant libraries limits the diversity and can produce hits that are difficult to optimize due to a lack of synthetically more accessible functionality [87,88] i.e., unavailability of structural information or lack of activity-determining features (scaffold with suitable functional groups). Moreover, fragments with a MW range of 150–300 Da that bind to the same site in close proximity can be further optimized into larger molecules with better binding affinities [89,90] by subsequent steps of linking, growing, and merging. 

Since the number of theoretical compounds increases exponentially with MW, smaller compounds enable a more efficient exploration of chemical space [91]. In addition, fragment molecules could easily bind a particular subsite within a binding site in comparison to a larger molecule that fits in size to the binding pocket [92].

Predesigned fragment libraries are becoming increasingly available directly from chemical vendors and Table 1 lists some of the commercial suppliers. These libraries offer a diverse range of collections which can be expanded with more targeted and novel sets of compounds. The commercial libraries provide a reliable, high quality, and cost effective diverse selection of compounds.

## 4. NMR Techniques for Screening

NMR is a versatile technique that can be used for screening, optimization, and validation of binding of a small molecule to its target macromolecule. Both ligand-based and target-based NMR spectra are extensively used in FBDD [95], yield typical throughput of 1–1000 compounds per screen, and require costly instrumentation [68]. Target-based methods, although very helpful, involve complex, expensive and time-consuming two-dimensional (2D) experiments with an isotopically labelled protein. Since structural information is usually required, a high level of backbone amide resonance assignment is necessary. To identify the binding mechanism the peaks in the ^15^N-HSQC data are assigned to every amino acid residue in the protein sequence. Ligand-based methods do not require structural information and involve rapid acquisition of one dimensional (1D) data. Ligand-based methods require much lower target molecule concentrations than target-based methods and work well for high molecular weight proteins. Ligand-based methods, such as STD, perform poorly on smaller proteins (<15–20 kDa) where target-based methods succeed [96]. Most ligand-based methods, however, provide no information about the ligand-binding site, which must be obtained from additional experiments. Target-based methods however, can actually be more informative than ligand based methods and relatively fast when small proteins are used [97]. Solution protein NMR spectroscopy is valuable for target-based drug discovery as it provides information on the target-ligand binding mechanism such as hit identification, ranking ligand binding affinities, and mapping the ligand binding site. In another example of target based methods, solid-state magic angle-spinning (MAS) NMR procedure is widely applicable to small membrane proteins expressed in bacteria [98]. Although numerous restrictions are imposed by the high molecular weight of target (around 40 kDa), there are several relaxation-optimized NMR techniques to tackle the relaxation and linewidth problems these days. Thus, making NMR a high throughput approach in hit generation and characterization [99].

**Figure 3:**
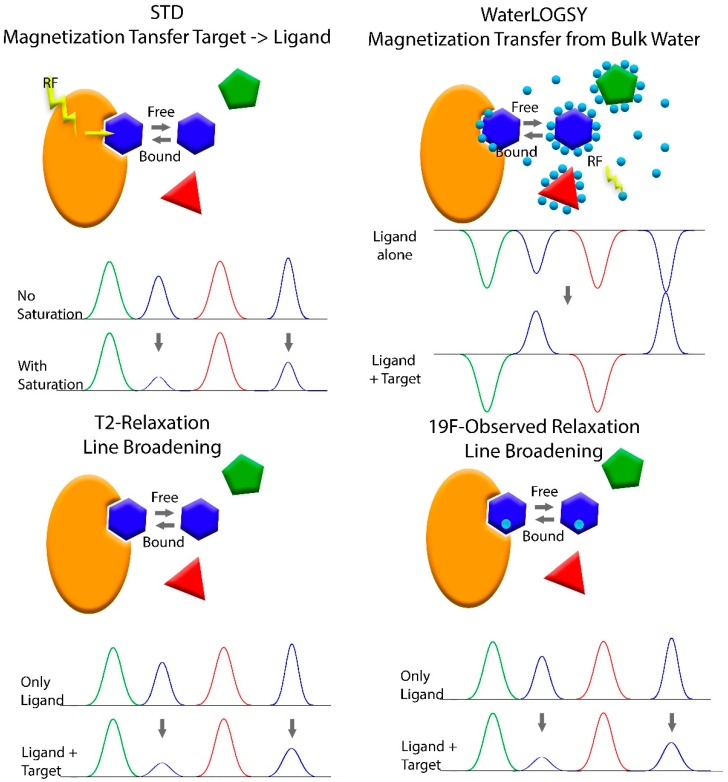
Schematic representation of different ligand detected methods used in fragment based screening.

Though several other biophysical methods are utilized for FBDD, NMR was the first and remains an important method for the discovery of new drugs. The first study on FBDD using NMR published by Shuker et al. in 1996 made use of chemical shift changes in 2D HSQC spectra of a protein to identify fragments that bind to the protein. Guided by NMR, relevant fragments were optimized, their binding site relative to each other determined and then fragments were linked to yield a high affinity ligand [8]. Using 2D spectra limits the method to relatively small biomolecules (<~40–60 kDa) at high concentrations, for obtaining high quality spectra in a reasonable amount of time. However, NMR is very versatile and since this pioneering study, several approaches for different stages of the drug discovery process have been applied for FBS. For the primary screen of a fragment library, one-dimensional ligand-detecting NMR methods are most commonly used, for several reasons: 1D spectra of small molecules are faster and easier to detect than 1D or 2D spectra of the target biomolecule. Using small molecules that yield uncrowded spectra avoids costly isotope labeling and even allows measuring samples containing several fragments at once. A major advantage of ligand-based detection methods is that these are not limited by the size of the target biomolecule and do not require a high concentration of the target. These ligand-based detection methods (schematically presented in Figure 3) exploit the differences in the physical properties of the ligand in its bound and free state. Although the measured signal originates from the unbound fragment, it still contains information from the bound state, in which the fragment behaves like a molecule of the size of the target rather than a small molecule, if the dissociation of the fragment from the target is within the timescale of the experiment.

The most widely used ligand-based method is the saturation transfer difference (STD) experiment [100]. This experiment uses a train of selective pulses to saturate signals of the protein that are in an area of the spectrum with no signals originating from the fragments (e.g., methyl protons of the proteins below 0 ppm). The saturation is transferred throughout the protein and to any bound fragments, causing a decrease in signal intensity. Comparing 1D spectra of the fragments with and without the saturation pulses reveals those fragments that bind to the protein. WaterLOGSY utilizes the sign inversion of the Nuclear Overhauser Effect of Water between the binary ligand bound state and the ternary state where the ligand is bound to the target protein [66,101]. This experiment also uses selective excitation, like the STD, however, instead of the protein the water is excited. Recent advances in NMR technology benefit from this experiment as well. The use of hyperpolarized water significantly increases the sensitivity. As protein signals are enhanced as well, this method can also be used to determine whether the protein is aggregated or intact and thus, eliminate false positives [102].

A ligand bound to a much larger target will adopt the relaxation properties of the large target in its bound state. A large protein tumbles at a much slower pace than a small molecule, which leads to much faster relaxation times. The Carr Purcell Maiboom Gill (CPMG) sequence can be used to determine the relaxation time T2 of the fragments in their free form and in a sample containing the target biomolecule. Fragments with reduced relaxation times are those that bind to the target [103,104]. The relaxation times is indirectly proportional to the line width at half height. Thus, shorter relaxation times of larger molecules lead to line broadening. Since the peak consists of a large contribution from the unbound state with a narrow line and a smaller contribution from the bound state with broad a line, the main effect observed in the 1D spectrum is a reduction in intensity. The large difference in relaxation times of the bound and unbound from can also be used to filter out resonances originating from the protein and the complex, for example by using a long echo time in the CPMG experiment or adding a filter to the STD experiment. Just as a ligand bound to a larger molecule will adopt the relaxation properties of that molecule, the ligand will also adopt the diffusion properties of the target and diffuse at a much slower pace than the free ligand. The diffusion rate of the free ligands and of the ligand in a sample containing the target molecule can be measured by NMR in an experiment called DOSY (Diffusion Ordered Spectroscopy) [104].

Sometimes spectral crowding and background noise from the biomolecule can be a hindrance. In this case, a ligand labeled with ^19^F might be a solution. ^19^F has only a slightly lower sensitivity than protons, but offers a much larger chemical shift range and eliminates the background from the target. The relatively high speed, low background signal/interference and ability to probe diverse pharmaceutical/environmental parameters in protein-observed ^19^F-NMR experiments helped to discover and characterize selective ligands for bromodomain-containing proteins [96]. Measuring the ^19^F relaxation rates of the free ligands and ligands in a sample with the biomolecule will identify those ligands that bind to the target by a shorter relaxation time, just like for the proton experiment [105].

Target-based methods require 2D experiments or even higher dimensionality and are thus more time consuming. Often isotope labeling of the target is required (for example ^15^N labeling for HSQC spectra, plus ^2^H labeling for larger proteins) and larger concentrations are required, making these experiments costlier. However, they do offer other advantages and are very useful for further characterization after an initial 1D screen identifying promising fragments. Target-based experiments can be utilized to obtain structural information at an atomic level, which is not available with any other technique. Titrating a ligand into a sample of the target molecule and measuring 2D HSQC spectra at each point allows the determination of the binding site of the ligand, as the chemical shift of the resonances involved in binding will change [106]. It should be noted, that chemical shift changes may also arise due to induced conformational changes at a site distant from the binding site or due to dimerization. Thus, chemical shift changes must be analyzed carefully. Changes that cannot be mapped to a single site, for example, are most likely due to conformational changes. Changes that can be mapped to a single site but are accompanied by line broadening could be due to dimerization, which could be confirmed by measuring relaxation properties. For more details see for example [106]. Depending on the binding affinity, the chemical shift of a protein resonance might gradually shift with increasing ligand concentration (slow exchange, tight binding) or gradually disappear and then reappear at the new position (slow exchange, tight binding). Titration experiments can also be used to determine binding constants for the ligand target interaction.

## 5. Optimization: Growing, Merging, and Linking Fragments into Potent Inhibitors

**Figure 4:**
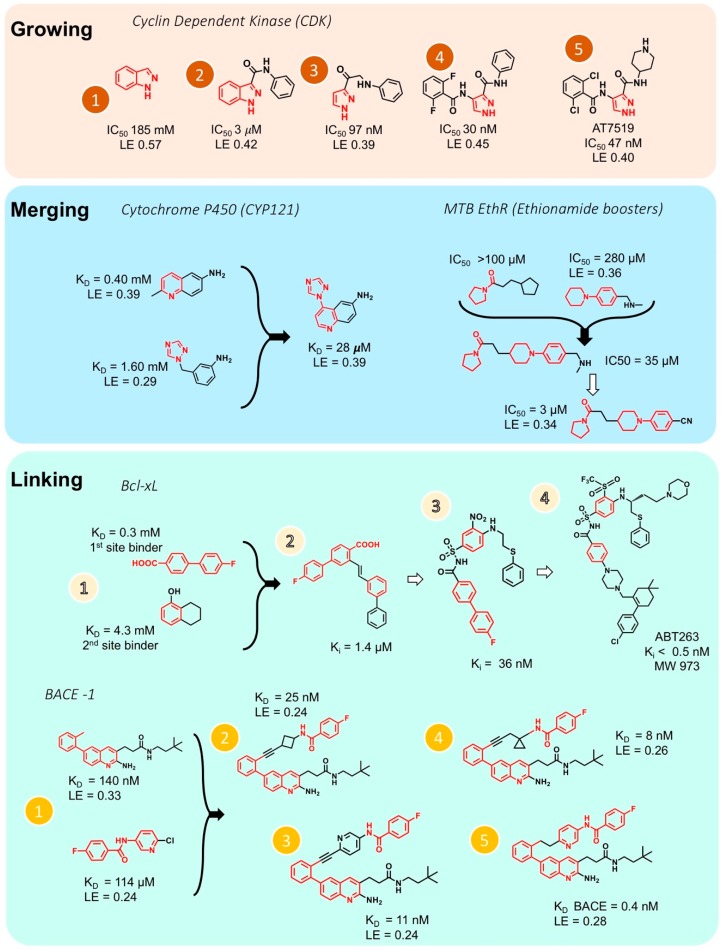
Different hit-to-lead optimization strategies (fragment growing, merging and linking approach). Upper: growing of fragments into inhibitor for cyclin dependent kinase (CDK) [107]. Middle: merging of fragments for inhibitors for cytochrome P450 (CYP121) [108], and *Mtb* EthR (Ethionamide boosters) [109]. Bottom: Linking of fragments into inhibitors for Bcl-XL [110] and β-secretase, BACE-1 [111]. KD, dissociation constant; LE, ligand efficiency; Ki, inhibition constant; IC_50_, concentration for 50% inhibition. Panel 1 (Growing) was adopted from Dan Erlanson’s blog (http://practicalfragments.blogspot.co.il/).

Fragment optimization to obtain a drug-like lead compound is an important step in FBDD. Unless the optimization of hits from a high throughput screen, which are larger in size and already have better binding affinities, fragments require extensive optimization through growing, merging and/or linking. Fragment growing is the easiest method of obtaining molecule with better binding properties by starting from a single fragment and extending its pattern of interactions with the target molecule using medicinal chemistry (Figure 4). However, atomic resolution structures obtained by crystallography or high-field NMR are essential for growing the fragment into a lead compound. One example for fragment growing is the drug AT7519 by Astex, an inhibitor of cyclin dependent kinase (CDK) [107]. Out of 500 fragments, 30 fragments possessing indazole moiety were identified that bind to the ATP binding site of CDK. Figure 4 (Growing panel) shows the development steps from the fragment indazole to the drug AT7519. In this case, fragment growing of the initial ‘indazole’ hit 1 led to a compound 2 with a 60-fold increase in potency. Removal of the phenyl ring of the indazole yielded a compound with an IC50 of 47 nM with only a small decrease in ligand efficiency (AT7519). AT7519 is currently in Phase II clinical trials and has shown good indications against a range of human tumor cell lines.

Two fragments that have some common structural features and bind to overlapping sites on the target but are otherwise different, can be merged to yield a more potent molecule. Figure 4 (Merging panel, left) shows the example of the development of an inhibitor of the mycobacterial tuberculosis cytochrome P450 CYP121 [108]. Two fragments with a similar phenylamine moiety were detected using X-ray crystallography. These two overlapping fragments were merged to yield an efficient inhibitor with 15–60-fold improvement of binding affinity comparing to the binding values of the two separated fragments. A more recent example for merging is also presented in Figure 4 (Merging panel, right) where two fragments containing 5 or 6 aza-membered non-aromatic heterocyclic moiety were systematically merged together using structural information from X-ray crystallography. The merged fragments yielded small molecule inhibitors which have 100-fold improvement in potency over the initial fragments [109].

If two fragments are identified that bind to slightly different sites of the target but are still close in space, these fragments can be linked, for example, by attaching a “bridge” between them, to obtain a larger molecule with better binding properties. Linking two fragments is a difficult task, as the orientation of the two fragments must be maintained exactly. Fesik and coworkers reported one of the first successful examples of fragment linking using NMR screening against apoptotic protein Bcl-XL (Figure 4, Linking panel, top) where the initial fragment linking using an alkene as the linker lead to a significant increase in potency [110]. Using a different linker led to the compound ABT263 with a K_i_ < 0.5 nM. This drug is currently tested in phase II clinical trials for the treatment of cancer. Recently, Judd and coworkers reported an example of fragment linking using ^19^F-NMR against the aspartic acid protease β-secretase (BACE-1, Figure 4, Linking panel, bottom), where the initial fragment linking with an alkyne gave a significant increase in potency [111]. Further elaboration led to the development of a new molecule which ultimately exhibits a more than 360-fold increase in potency while maintaining reasonable ligand efficiency. However, in several studies dockings has been utilized following fragment screening to obtain drug-sized molecules [112,113].

### 5.1. Using NMR to Guide the Optimization of Fragments

NMR provides not only powerful methods for the screening stage, but can also be utilized for the optimization of the fragments. Although it can be used at any stage and for any of the described optimization methods, the use Structure-Activity relationships (SAR) by NMR is especially popular. SAR by NMR was first described by Shuker et al. in 1996 [8] and is based on NMR-guided optimization and linking of two fragments that bind to subsites of the target molecule. After identifying a first fragment through screening, the library is screened again with saturating concentrations of the first identified fragment to be able to identify fragments that bind near the binding site of the first fragment. The scientists in the original study mainly used 2D ^15^N-HSQC target detected spectra to develop an inhibitor for the immunosuppressant FK506. Target detected spectra are required to be able to screen for fragments binding near each other, which would not be possible with 1D spectra. However, target detected spectra are limited to proteins up to a certain size and require the assignment of the protein resonances. NMR techniques that do not require the assignment of the target molecule are often based on the Nuclear Overhauser Effect (NOE). One popular method is NOE matching, in which the experimental NOE data is compared to NOE data of predicted binding positions of the small molecule to the target to identify the actual binding position [114]. Another is SAR by ILOEs (Inter ligand NOEs) in which NOE interactions between the bound fragments are detected directly [115]. ILOEs provide information about the orientation and distance of the fragments to each other, which is important information for creating a linker. As SAR by NMR enables the development of highly potent and specific compounds it continues to be one of the most popular and successful NMR techniques for FBDD [116,117,118,119,120]. There have been other remarkable examples where SAR by NMR was used as a primary optimization technique to find potent inhibitors such as Bcl-2 [121] and HSP90 [122] inhibitors. Abbott laboratories developed an inhibitor of Bcl-2 family proteins using NMR-based screening, parallel synthesis and structure-based design. ABT-737, a small-molecule inhibitor of the apoptotic proteins Bcl-2, Bcl-XL and Bcl-w, with improved potency were shown to induce regression of solid tumor. Hajduk and co-workers reported the discovery of novel HSP90 inhibitors using a multiple fragment based design approaches for the treatment of cancer [122]. They developed two initial hits which intriguingly linked together using a fragment linking approach, to yield novel inhibitors with micromolar range activities. Design of linking chemistry is challenging as most fragments are expected to bind into the same binding cavity. The linking, however, is expected to retain all chemical bonds of the newly larger molecule with the target without altering or affecting the position, orientation, or the bonding with the target of the two individual fragments.

Another option for target detected optimization is the use of fluorinated target proteins. The introduction of selected ^19^F labels into the protein provides a probe with high sensitivity and significantly reduces overlap and enables target detection of larger proteins. Although care must be taken that the modification of the protein does not alter the structure and function of the protein nor the binding of the fragment to the binding site, this method offers a promising approach [114,123].

### 5.2. Virtual Screening and Virtual Filtration

Some important questions persist on the fundamental and the practical aspects of growing/merging and linking of fragment hits. Two of these aspects regard the utility of molecular docking for prioritizing fragments, and the specificity of fragment inhibitors towards potent molecules. The low-throughput nature of FBS as well as the tedious optimization procedure that follows makes computational docking of drug-sized molecules an attractive tool to prioritize fragments from the much larger commercially available dataset. Only hundreds to thousands of fragments can be screened using most fragment screening techniques in a single screening trial, whereas, more than 250,000 fragments are commercially available [124] leaving a large portion of fragment libraries untested. Since commercially available fragments are too numerous to be screened experimentally, complementary conventional tools can be advantageous. Computational chemistry tools are used to explore larger commercially available fragment databases and can significantly improve the efficiency of the individual steps of FBDD, such as fragment library design, active site categorization, fragment hit discovery, and hit-to-lead-to-candidate optimization [125]. Moreover, many recent reviews are available that discerningly and comprehensively compare docking methodologies, scoring functions and their wide applications in drug discovery [126,127,128].

There are many databases used for virtual screening of drug-sized molecules (not fragments, Table 2) some of which are collections of commercially available compounds, such as ZINC [124]. In addition, most pharmaceutical companies typically maintain their own internal database of previously synthesized compounds. An alternative to the commercial collection is the open NCI database [129], a set of compounds that have been screened for anticancer activity over the past few decades and for research purposes subsets of this collection are available upon request for the virtual screening (http://dtp.nci.nih.gov/branches/dscb/repo_open.html).

A report by Peach et al. [130] describes a combined approach of docking with pharmacophore filtering for improved virtual screening. The relatively simple method for reducing the number of false positives was developed in order to filter out the ligands with high rank order by virtual screening. In fact, this technique uses a docking program for pose generation only, irrespective of scoring functions, followed by receptor-based pharmacophore filtering.

## 6. Successful Attempts of Molecules Derived from FBDD

In the past decade, FBS has become a successful approach for developing new inhibitors against complex targets. The anti-melanoma drug vemurafenib, a selective inhibitor of B-Raf kinase was the first FDA-approved drug discovered by employing high concentration screening (HCS) and FBS using X-ray crystallography [131]. Thereafter, Bcl-2 inhibitor venetoclax has been approved for the treatment of chronic lymphocytic leukemia [132], originated from NMR screening [110]. Recently, LEE011 (also known as Ribociclib or Kisqali), a selective cyclin dependent kinase inhibitor (CDK4/6) that was developed by the Novartis Institutes for BioMedical Research (NIBR) in collaboration with Astex Pharmaceuticals has been approved by the FDA. FBDD led to the development of LEE011 using structure-guided drug discovery and the crystal structure of the cancer target CDK4 [133]. It received the FDA approval in combination with an aromatase inhibitor letrozole [51] as a first-line treatment in post-menopausal women with hormone receptor positive, human epidermal growth factor receptor-2 negative (HR+/HER2−) advanced (metastatic) breast cancer.

Concomitantly, the low throughput nature of fragment testing makes computational methods such as docking, a suitable option to prioritize fragments from the large commercially available dataset. Certainly, numerous groups have used docking to prioritize fragments for testing [112,113]. Recently, Spiliotopoulos and co-workers presented high throughput docking of fragment molecules to the N-terminal bromodomain of the Bromodomain containing protein 4 (BRD4) and the cAMP-response element-binding protein (CREBBP) bromodomain using anchor-based library tailoring (ALTA, mentioned in Section 2) [134]. Some examples using a combined approach of FBDD with virtual screening which have led to the development of potent inhibitors based on optimized fragments are summarized in Table 3 and Table 4.

## 7. Recent Improvements: Fragment Based Virtual Screening (FBVS)

**Figure 5:**
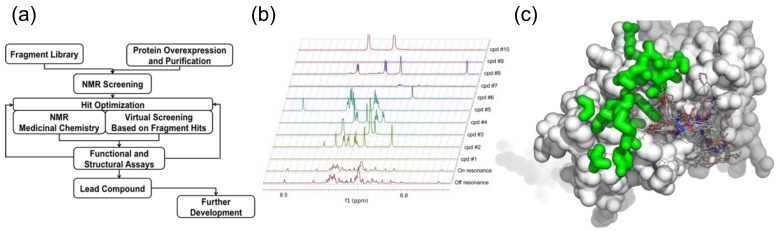
NMR Fragment-based virtual screening. (**a**) Schematic representation of FBVS. The approach combines NMR-FBS with optimization steps using virtual screening; (**b**) Using NMR (STD) and a fragment library, fragment molecules that bind a protein target are identified. Every experiment involves mixing of 10 fragment molecules and T7 primase (off/on resonance indicate spectra of fragments mixture). The difference between the off to the on-resonance spectra is the STD. The fragment molecules represent scaffolds for the next step of virtual filtration, i.e., using virtual filter to select larger compounds containing the fragment molecules from a database of multimillion drug-like molecules; (**c**) Thousands of drug-like molecules for each scaffold are then, using docking software, inserted into a targeted binding site using the atomic resolution structure of the target macromolecule. Hits are ranked on the basis of the binding energy. Ten to twenty candidate compounds are then selected and tested for their ability to inhibit the biochemical target.

The basis for FBS is that binding of functional chemical groups to the target can build up a drug-like molecule [159,160]. T7 primase, an essential protein domain encoded by the bacteriophage T7 gene 4-helicase-primase [161], was chosen as a drug target to select small-molecule inhibitors of DNA replication using FBS. T7 DNA primase is a slow enzyme displaying a rate constant of ~4 s^−1^ [162]. The weak catalytic activity of DNA primase renders a formidable challenge of adapting a functional assay to HTS, and therefore was an ideal candidate for FBS. T7 primase is also an excellent model for bacterial primases [161] that serve as novel targets for a new class of antibiotics [163,164,165]. We have combined FBS and virtual screening (FBVS, Figure 5) to select small molecules that target the bacterial primase. Specifically, by using the Maybridge Ro3 fragment library, composed of 1000 fragments, we have prepared 100 NMR samples containing a mix of 10 fragments and 50 μM T7 primase each. The 1D Saturation Transfer Difference (STD) spectra of these samples were measured and fragments showing saturation transfer were identified by a decrease in the peak intensity at specific chemical shift values. The hits were ranked based on the number of peaks affected and by the percentage of intensity change. The indole and methyl quinoline-6-carboxylate fragments were the highest-ranking ones. We then shortened the lengthy optimization process by searching the ZINC database [166], which contains the structures of tens of millions of compounds. In this virtual filtration step, drug-like molecules containing fragment molecules found at first by NMR-FBS were selected. This search yielded a few hundred to a few thousand molecules per fragment-molecule. The computer program Autodock [167] was then used to perform in-silico docking of those compounds to the active site of the T7 primase by using its crystal structure [168] (PDB ID 1nui). The drug-like molecules were ranked based on their relative binding energies and the top 18 small molecules from this list were purchased. Five of the drug-like molecules were found to inhibit T7 DNA replisome through specific inhibition of DNA primase. The binding of the small molecules identified using FBVS was validated using [^15^N, ^1^H] TROSY HSQC spectra of ^15^N, D labeled T7 primase in the absence and upon binding of selected small molecules and a mechanism of binding was proposed [161].

## 8. Summary and Future Directions

In the last few years fragment based screening has becoming a main stream approach in drug discovery, and has thus far yielded ~30 drug in various stages in the clinical pipeline. 

The combination of FBS with computational techniques such as virtual screening and docking allows to benefit from these different approaches. Each contributes specialized advantages and together bring genuine complementation that can build a better inhibitor. FBVS is one example for utilizing NMR-fragment based screening with virtual screening to gain a high success rate in a rapid, inexpensive manner, without the need for intervention of medicinal chemistry in the early stage to grow the fragment molecules into larger, more potent drug-sized inhibitors. The proof-of-concept study of FBVS on T7 primase serves as a basis for the development of lead molecules against other drug targets. Target selection should not be limited to proteins but can direct the search of fragments toward other macromolecules such as nucleic-acids, depends on the NMR method used for screening of the fragment molecules in the first step of FBVS. The modular arrangement of FBVS not only allows to change the first step of fragment screening but also to modify the later steps of virtual filtration and docking based on available improved resources.

We envision that in the future, FBS-NMR will become more popular in drug discovery and will yield potent inhibitors for popular drug targets including G-protein coupled receptors, nuclear receptors, ion channels or enzymes (e.g., kinases, ATPase, proteases, deacetylases, etc.). With the advance of computer aided-techniques in drug design and enlargement of small molecule libraries, the modular nature of FBVS will be updated with any technological advancement. Specifically, we believe that analysis of FBS can become automatic to yield novel lead molecules for drug target even those that were traditionally considered as “non-druggable” or challenging such as protein-protein interactions. The use in NMR as a tool for screening but also for optimization and validation can provide detailed plan for SAR cycles that will allow to add onto the pre-existing small molecule inhibitors improvements to design larger spectrum medicines or alternatively inhibitors with larger selectivity. The ability of NMR to provide detailed knowledge of the binding site and mode can then be used to build up a better drug-like molecule on the basis of the fragment hit. The use of NMR technique for screening is not limited to size of the macromolecular target, however, in the optimization and validation steps (after fragment molecules were found in the initial screening) protein target size is limited up to 40 kDa and in turn requires the assignment of backbone and side-chain resonances to elucidate the complete binding information of the small molecule. For example, NMR was used to identify novel allosteric ‘hot spots’ on traditionally targeted proteins such as those present in protein kinases and intrinsically disordered proteins [54].

Large molecular weight targets comprising multi-subunit protein complexes pose a major limitation mainly for NMR spectroscopy but also to X-ray spectroscopy. In the hit-to-lead optimization phase structural data is the rate limiting step to monitor rationality for compound expansion and introduce new chemical alterations. Prior information of the target structure doesn’t mean that small molecule hit binding will be easily validated structurally. In order to use NMR as a complementary technique to provide means for structural information at atomic resolution for these types of targets, NMR active isotope labelling and multidimensional experiments is necessary. Size remains the main limitation, however multiple labelling schemes and experiments are readily available, making it now possible to handle assemblies as big as the 1 mega Dalton proteasome complex [169,170].

## Figures and Tables

**Table 1:** List of commercial suppliers of fragment libraries.

Commercial Supplier	Library Name	Number of Fragments	Remarks
ACB Block	^19^F-NMR-oriented Fragment library	1280	Ro3 compliant
	Fragment Library for NMR	760	Ro3 compliant
Asinex	Fragment library Building blocks	>22,000	Modified Ro3
Analyticon	Fragments from natural products	5000	FRGx: fragments from
			nature; Ro3 compliant
ASDI	Fragment screening collection	1700	Ro3 compliant
	Diversity fragment library	6800	Ro3 compliant
BIONET	Fluorine Fragment Library	461	Ro3 compliant and substructure filtering by PAINS
Biofocus	BioFocus’ 3D-biased fragment sets	1500	Modified Ro3
			Surface plasmon resonance (SPR) screening used [ 93]
ChemBridge	Fragment library	>7000	Ro3 compliant
	ChemBridge Microformat Library	20,000	Ro3 compliant
Charles River	Core fragment library	1500	Modified Ro3 compliant
	Kinase focused fragment library	500	
	^19^F labeled fragment library	500	
ChemDiv	3D designed fragment library	>4000	Ro3 compliant
Enamine	“Simple” fragment library	126,597	Ro3 compliant ≤20 heavy atoms from screening collection
	Ro3 Fragment Library	44,600	
	sp3 Rich Fragment Libraries	14,000	
	PPI Fragment Library	3500	
	Fluorinated Fragment Library	2100	
	Brominated Fragment Library	1200	
	Covalent Fragment Libraries	3000	
	Essential Fragment Library	190	
	Single Pharmacophore Fragments	3200	
	Carboxylic Acid Fragment Library	4300	
	Golden Fragment Library	1794	
InFarmatik	Consolidated library with different subsets (diverse 3-D fragments, GPCR, kinase)	1700	Ro3 compliant
IOTA Pharmaceuticals	Fragment library	1500	mainly Ro3 compliant fragments
Key Organics	Fragment library	~26,000	multiple subsets with assured solubility and Ro3 compliant
	2nd generation subsets	1166	Ro3 compliant
			assured aqueous solubility
	Fragments from nature	183	Ro3 compliant, assured solubility and high Fsp3 content
	CNS fragment library	700	
	Brominated library	1656	
	Fluorinated fragments	1950	
Life Chemicals	Multiple subsets	31,000	Brominated, covalent, Fsp3-enriched, and covalent subsets.
			14,000 of which are Ro3 compliant


	Diversity Fragments set	3500	
	^19^F-Fluorine-Based Fragment Library	1300	
Maybridge (Thermo-Fischer)	Fragment library for NMR	>30,000	Ro3 compliant
			Dedicated to NMR applications. A smaller 1000-fragment subset is also available, probably to identify more hits [ 94]
	Diversity Fragment Library	2500	Latest addition
			Ro3 compliant
			Guaranteed soluble at 200 mM in DMSO and 1 mM in PBS
	Maybridge Bromo-Fragment Collection	1500	Fragment library for X-ray based fragment screening
	Maybridge Fluoro-Fragment Collection	5300	Fragment library for ^19^F-NMR based applications
Otava	Fragment library	Total of 12,486	General Ro3 compliant



	Chelator Fragment Library	1023	
	Halogen-Enriched (Bromine) Fragment Library	618	
			used for X-ray crystallography based fragment screening
	OTAVA’s ^19^F-NMR Fluorine-containing Fragment Library	1077	
Prestwick	Fragment Library	2230	Ro3 compliant
			Contains set of known drugs MW < 300, together new 910 fragments derived from drug molecules
Pyxis	Fragment Library	317	Based on scaffolds that are found in existing drugs
TimTec	Structurally diverse fragment library	3200	Modified Ro3
Vitas-M	Commercial fragment library	18,932	Ro3 compliant
Zenobia Therapeutics	Commercial fragment library	968	Ro3 compliant
			Fragments derived from different design paradigms, cores from drugs, higher Fsp3, flexible cores

Note: Fragment library in bold can be used exclusively for NMR based applications.

**Table 2:** Virtual Screening libraries.

Libraries Used for Virtual Screening	Library Name	Number of Compounds	Remarks
ZINC http://blaster.docking.org/zinc/	Total purchasable	35,724,825	Free database of commercially-available compounds. Compounds available in ready-to-dock, 3D formats.
	Drug like	17,900,742	
	Fragment like	847,909	
	Lead like	6,053,287	
ChemNavigator iResearch Library (www.chemnavigator.com)	iResearch Library	>160 million chemical samples	Paid library Commercially accessible screening compounds from international chemistry suppliers
National Cancer Institute (NCI) https://dtp.cancer.gov/organization/dscb/obtaining/default.html	NCI/Developmental Therapeutics program (DTP) Open Chemicals Repository	>200,000	Compounds available free of charge
MDL Inc. http://www.iop.vast.ac.vn/theor/conferences/smp/1st/kaminuma/ChemDraw/acd.html	Available Chemicals Directory (ACD)	351,600 3D models	Paid library Compounds available in 3D models
CCDC’s Cambridge Structural Database http://www.ccdc.cam.ac.uk/products/csd/	Cambridge Structural Database	over 900,000 entries	Repository for small-molecule organic and metal-organic crystal structures Highly curated and comprehensive resource of unique database with accurate 3D structures
PubChem http://pubchem.ncbi.nlm.nih.gov/	Pubchem substance Pubchem compound PubChem BioAssay	234,688,140 93,553,459 1,252,796	Three databases of PubChem are linked within the NCBI’s Provides a fast-chemical structure similarity search tool

**Table 3:** Fragment derived compounds in clinical stage of development representing method of detection.

Drug & Ref.	Company	Target	Status	Method of Detection
LEE011 (ribociclib) [51]	Novartis/Astex	Cyclic dependent kinase CDK4/6 (Breast cancer)	Approved	X-ray Crystallography
Vemurafenib [131]	Plexxikon	B-Raf-V600E (metastatic melanoma)	Approved	HCS/X-ray
Venetoclax [50]	AbbVie/Genentech	Selective Bcl-2 (recalcitrant chronic lymphocytic leukaemia)	Approved	Target-detected NMR
PLX3397 [135]	Plexxikon	FMS, KIT, and FLT-3-ITD (Cancer)	PhaseIII	Functional assays/X-ray Crystallography
Verubecestat (MK-8931) [136,137]	Merck	BACE1 (Alzheimer’s disease)	PhaseIII	NMR/Crystallography
AZD3293 (lanabecestat) [138]	AstraZeneca/Astex/Lilly	BACE1 (Alzheimer’s disease)	PhaseIII	X-ray crystallography/NMR/calorimetry
AT7519 [139]	Astex	CDK1,2,4,5,9 (Multiple myeloma)	PhaseII	X-ray Crystallography
AT9283 [140]	Astex	Aurora, JAK2 (Multiple myeloma)	PhaseII	X-ray Crystallography
AT13387 [141]	Astex	HSP90 (gastrointestinal stromal tumours)	PhaseII	Ligand-detected NMR/Crystallography
NVP-AUY922 [142]	Vernalis	HSP90 (cancer)	Phase II	Ligand-observed NMR screening
AZD5363 [143]	AstraZeneca/Astex/CR-UK	AKT Serine threonine protein kinase (Cancer)	PhaseII	X-ray Crystallography
Erdafitinib (JNJ-42756493) [144]	J & J/Astex	FGFR1-4 (Cancer)	PhaseII	X-ray Crystallography
Indeglitazar [145]	Plexxikon	pan-PPAR agonist (Type II Diabetes melitus)	PhaseII	HCS/X-ray Crystallography
LY2886721 [146]	Lilly	BACE1 (Alzheimer’s disease)	PhaseII	Co-crystallization
LY517717 [147]	Lilly/Protherics	FXa (thrombotic)	PhaseII	X-ray Crystallography
Navitoclax (ABT-263) [148]	Abbott	Bcl-2/Bcl-xL (Cancer)	PhaseII	NMR
NVP-AUY922 [149]	Vernalis/Novartis	HSP90 (Breast cancer)	PhaseII	X-ray Crystallography
Onalespib (AT13387) [141]	Astex	HSP90 (Cancer)	PhaseII	NMR/X-ray Crystallography
AT9283 [140]	Astex	Aurora (Cancer)	PhaseII	X-ray Crystallography
ABL001 [150]	Novartis	BCR-ABL 1 (Chronic myeloid leukaemia)	PhaseI	X-ray Crystallography
ABT-518 [151]	Abbott	MMP-2 & 9 (Cancer)	PhaseI	SAR by NMR/ LCMS/Mass spectrometry
DG-051 [152]	deCODE	LTA4H (cardiovascular and inflammatory)	PhaseI	X-ray Crystallography
IC-776 [153]	Lilly/ICOS	LFA-1 (autoimmune diseases)	PhaseI	NMR
PLX-4032 [131]	Plexxikon	B-RafV600E (metastatic melanoma)	PhaseI	HCS/X-ray
PLX5568 [154]	Plexxikon	Raf kinase (Polycystic Kidney Disease)	PhaseI	HCS/X-ray
SGX-523 [155]	SGX	Met tyrosine kinase (Tumour)	PhaseI	X-ray/HCS
SNS-314 [156]	Sunesis	Aurora kinase (Cancer)	PhaseI	Mass Spectrometry

Note: Some of the information of the Table 3 was adopted from Dan Erlanson’s blog (http://practicalfragments.blogspot.co.il/).

**Table 4:** Recent examples of FBS that yielded lead molecules.

Entry & Ref.	Target	Methods	Library	Fragment	Lead	Lead IC_50_ (nM)
1 [157]	Plm * I, II and IV of Plasmodium parasites (malaria)	NMR STD	ChemBridge containing 976 Astex Ro3 compliant compounds	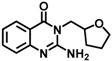	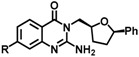	plmI, 10,000 plmII, 3200 plmIII, 130
2 [114]	BACE1 *	^19^F-NMR SPR	^19^F fragment library	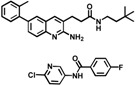	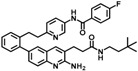	0.8
3 [158]	IRAK4 * autoimmune diseases	STD NMR and biochemical assays	Global Fragment Initiative library 2592 fragment	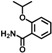	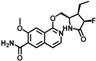	55

* Plm-plasmepsin, BACE1-β-secretase, IRAK4- Interleukin-1 Receptor Associated Kinase 4.
